# Association between knowledge of cervical cancer prevention and screening behaviors among women aged 20 to 49 years: a cross-sectional study in six provinces, China

**DOI:** 10.1186/s12889-025-22971-2

**Published:** 2025-05-17

**Authors:** Di Gao, Xueyin Wang, Juan Juan, Zhifei Pei, Xiaosong Zhang

**Affiliations:** 1 Department of Obstetrics and Gynecology, Peking University First Hospital, Beijing, 100034 China; 2 Department of Obstetrics and Gynecology, Xicheng Maternal and Child Health Care Hospital, Beijing, 100054 China

**Keywords:** Cervical cancer, Knowledge, Screening, Reproductive-age women, HPV

## Abstract

**Background:**

Cervical cancer is regarded as the fourth most common cancer in terms of both incidence and mortality among women worldwide. Cervical cancer screening is a crucial method to achieve early diagnosis and treatment of cervical intraepithelial neoplasia and cervical cancer. The screening behaviors among women have been linked to knowledge level of cervical cancer prevention, yet little is known about the association in various areas and regions of China.

**Methods:**

A cross-sectional study was conducted from June to September 2018 in six provinces of China. In this survey, knowledge level of cervical cancer prevention was assessed by a set of 7 question items, including the awareness, risk factors, preventive actions of cervical cancer, as well as awareness and effects of HPV vaccines, and also the benefits of regular cervical cancer screening. Screening behavior was determined by asking women whether they have had ever participated in cervical cancer screening. Socio-demographic characteristics were collected by questionnaire. Multivariate logistic regression models were used to analyze the association between cervical cancer screening behaviors and knowledge level.

**Results:**

A total of 9144 women aged 20–49 years were involved in the analysis, with an average age of 37.9 ± 8.5 years. There were 37.6% of participants reported having ever screened for cervical cancer. The rate of cervical cancer screening behaviors was significantly associated with region, area, age group, occupation, education level, marital status, gravidity and knowledge level. Women with a high level of knowledge (score ≥ 5) were more likely to have screening behaviors than those with a low knowledge level (OR = 2.91, 95% CI: 2.63–3.21). Compared to women in the knowledge score ≤ 1 group, the screening rate of women with the knowledge score ≥ 6 significantly increased regardless of the regions (western region: OR = 19.62, 95% CI: 12.39–31.04; central region: OR = 10.09, 95% CI: 6.76–15.06; eastern region: OR = 5.23, 95%CI: 3.62–7.56) and areas (urban area: OR = 12.70, 95% CI: 8.79–18.36; rural area: OR = 7.12, 95%CI: 5.19–9.77).

**Conclusions:**

Overall, our study demonstrated that the screening rate and knowledge level of cervical cancer among Chinese women still need to be improved. There was a significant association between knowledge scores and screening rates, regardless of region or area. Therefore, it is necessary to enhance the knowledge level of cervical cancer through intervention measures in order to promote regular cervical cancer screening.

## Introduction

Cervical cancer, the fourth most frequently diagnosed cancer and the fourth leading cause of cancer death in women, is an important public health issue of global concern, particularly in low- and middle-income countries [1]. An analysis of 700 population-based cancer registries of China estimated that approximately 150,700 cases of cervical cancer and 55,700 deaths in 2022 [2]. The incidence and mortality of cervical cancer are expected to decrease in the future with application of human papillomavirus (HPV) vaccines and cervical cancer screening. Although the upward trend of the young generations in urban areas is starting to slow down or even reverse, the disease burden of cervical cancer in China is still rising [3]. In particular, there are substantial regional variations in HPV vaccination coverage [4] and cervical cancer screening coverage [5].

The World Health Organization (WHO) set a 2030 target of 70% cervical cancer screening coverage for women aged 35–45 years, which is a crucial monitoring indicator of the WHO cervical cancer elimination plan [6]. China implemented a national free cervical cancer screening program in 2009, with a specific emphasis on rural women aged 35–64 years. Furthermore, China launched the accelerated action plan for cervical cancer elimination (2023–2030) in 2023, with the aim of achieving a 50% cervical cancer screening rate among eligible women by 2025, and ultimately reaching a target of 70% by 2030. According to a nationally and provincially representative survey data in 2018–2019, cervical cancer screening coverage in China reached 43.4% in women aged 35–44 years and 36.8% in women aged 35–64 years [5].

Previous studies have demonstrated that cervical cancer screening adherence among women was associated with marital status, educational attainment, having healthcare, smoking, physical activity, parity, chronic disease, and obesity [7]. Additionally, the level of knowledge related to cervical cancer also plays a crucial role in promoting preventive behaviors, including participation in screening programs [8]. Adequate knowledge about cervical cancer risk factors, symptoms, and prevention methods empowers women to make informed decisions and take preventive actions. However, findings from various studies conducted in specific cities in China have consistently revealed a relatively low level of knowledge regarding cervical cancer, HPV, and HPV vaccines among women [9–11]. This lack of knowledge may contribute to low screening rates and delays seeking appropriate healthcare [12].

Understanding the association between knowledge of cervical cancer prevention and screening behaviors among women is crucial for the development of effective public health interventions aimed at improving cervical cancer screening. However, limited research has explored this association in the general female population in Chinese context, particularly within different regions and areas. Nevertheless, investigating the factors that influence screening practices could help provide insights into the barriers and facilitators of cervical cancer prevention, and find out prioritized target population for potential interventions.

This cross-sectional study aimed to explore the association between knowledge level of cervical cancer and screening behavior among women in six provinces, China. Moreover, whether regions and areas might affect the association between knowledge level of cervical cancer and screening behavior was also assessed in this study.

## Methods

### Study design and sampling

The cross-sectional, community-based study was conducted from June to September 2018 in six provinces representing three socio-economic regions of China: eastern (Jiangsu and Shandong provinces), central (Hunan and Anhui provinces), and western (Shaanxi and Sichuan provinces). The capital city of each province, including Nanjing, Jinan, Changsha, Hefei, Xi’an, and Chengdu, was selected as a representative city. Within each city, one urban area and one rural area were randomly chosen as the survey sites. Details of this study have been described elsewhere [13, 14].

A multi-stage stratified random cluster sampling approach was employed to recruit participants for the study. A total of 490 women in each age group (20–39 years and 40–49 years) were included in the sample at each investigation site. Face-to-face interviews were conducted by community health service workers to collect information on demographic characteristics, knowledge of cervical cancer prevention, and also cervical cancer screening practice. These investigators, who had undergone comprehensive training on the study protocol and questionnaire investigation procedures, were obligated to conduct a thorough review of the filled-in content after the questionnaire was completed by participants. A total of 9144 women aged 20–49 years were involved in the analysis, with an average age of 37.9 ± 8.5 years. All participants provided written informed consent, and the study has been approved by the Ethical Review Committee of the Chinese Center for Disease Control and Prevention (IRB 201810).

## Data collection

In this survey, women were required to estimate cervical cancer and HPV related knowledge using a set of 7 question items. The items in the self-designed questionnaire were developed based on the key messages for cervical cancer education presented in the guide issued by the WHO [15]. These items covered various aspects, including awareness, risk factors, preventive actions of cervical cancer, as well as awareness and effects of HPV vaccines, and also the benefits of regular cervical cancer screening. Among them, risk factors and prevention methods for cervical cancer were composed of seven items, and correctly identifying more than half (≥ 4) of the seven questions was considered as correct. To quantify their level of knowledge, one score was assigned for each correct answer, resulting in a knowledge score ranging from 0 to 7 for each participant. Participants were categorized into two levels of knowledge: low level (score < 5), and high level (score ≥ 5), according to the median of the knowledge score in our study population. In addition, screening behavior was assessed by asking “Have you ever participated in cervical cancer screening?”

To account for potential confounding factors, several socio-demographic characteristics were included as covariates in the analysis. Covariates included socio-demographic characteristics including region (eastern/central/western), area (urban/rural), age, occupation, education level, monthly family income, marital status (unmarried/married/others), gravidity (number of pregnancies), age at menarche, etc.

### Statistical analysis

The categorical variables were presented by numbers (n) and percentages (%). The Chi-square test was applied to identify differences in socio-demographic factors, cervical cancer-related knowledge between the ever screened and never screened groups. Univariate and multivariate logistic regression models were used to analyze the factors associated with cervical cancer screening behaviors, and the association between knowledge scores and screening behaviors. Odds ratios (ORs) and 95% confidence intervals (CIs) were calculated. All fully adjusted models were adjusted for potential confounding factors, including region, area, age, occupation, education level, monthly family income, marital status, gravidity and age at menarche. In addition, stratified analyses were further conducted to explore the relationships within different regions and areas. As a sensitivity analysis, logistic regression models were performed specifically for women aged 35–49 years. Statistical analyses were performed using STATA 14.0 (Stata Corporation, College Station, TEXAS, USA). All tests were two sides, with *P* < 0.05 considered to be statistically significant.

## Results

### Characteristics of study participants

Among 9144 women aged 20–49 years included in the study, 37.6% (3438/9144) of participants reported having ever screened for cervical cancer. Characteristics of the study participants were shown in Table 1. Compared to women who had never received cervical cancer screening, those with screening experience were more likely to come from the eastern region, be in older age groups, have employment, higher educational levels, higher income, be married, have a history of pregnancies, and experience a later age at menarche (*P* < 0.05).

**Table 1:** Socio-demographic characteristics of participants who have never and ever been screened for cervical cancer

Variable	Total (*N* = 9144)	Never screened (*N* = 5706)	Ever screened (*N* = 3438)	*P*
Region				< 0.001
Eastern	3111 (34.0)	1788 (31.3)	1323 (38.5)	
Central	2989 (32.7)	1784 (31.3)	1205 (35.0)	
Western	3044 (33.3)	2134 (37.4)	910 (26.5)	
Area				0.088
Urban	4520 (49.4)	2781 (48.7)	1739 (50.6)	
Rural	4624 (50.6)	2925 (51.3)	1699 (49.4)	
Age group (y)				< 0.001
20–24	875 (9.6)	805 (14.1)	70 (2.0)	
25–29	1238 (13.5)	965 (16.9)	273 (7.9)	
30–34	1283 (14.0)	785 (13.8)	498 (14.5)	
35–39	1177 (12.9)	635 (11.1)	542 (15.8)	
40–44	2111 (23.1)	1150 (20.2)	961 (28.0)	
45–49	2460 (26.9)	1366 (23.9)	1094 (31.8)	
Occupation				< 0.001
Managerial and technical staff	1583 (17.3)	875 (15.3)	708 (20.6)	
Commercial/service personnel	1557 (17.0)	962 (16.9)	595 (17.3)	
Workers or farmers	4196 (45.9)	2523 (44.2)	1673 (48.7)	
Students	285 (3.1)	266 (4.7)	19 (0.6)	
Unemployed	1197 (13.1)	876 (15.4)	321 (9.3)	
Others	326 (3.6)	204 (3.6)	122 (3.5)	
Education level				< 0.001
Primary school and below	1748 (19.1)	1240 (21.7)	508 (14.8)	
Middle school	3051 (33.4)	1810 (31.7)	1241 (36.1)	
Senior high school or equivalent	1876 (20.5)	1108 (19.4)	768 (22.3)	
College and above	2469 (27.0)	1548 (27.1)	921 (26.8)	
Monthly family income (RMB)				< 0.001
<3000	2438 (26.7)	1632 (28.6)	806 (23.4)	
3000–4999	2855 (31.2)	1810 (31.7)	1045 (30.4)	
5000–7999	2241 (24.5)	1333 (23.4)	908 (26.4)	
≥8000	1610 (17.6)	931 (16.3)	679 (19.7)	
Marital status				< 0.001
Unmarried	1013 (11.1)	928 (16.3)	85 (2.5)	
Married	7784 (85.1)	4569 (80.1)	3215 (93.5)	
Divorced/Widowed/Others	347 (3.8)	209 (3.7)	138 (4.0)	
Gravidity				< 0.001
0	1292 (14.1)	1126 (19.7)	166 (4.8)	
1	2847 (31.1)	1633 (28.6)	1214 (35.3)	
2	2865 (31.3)	1665 (29.2)	1200 (34.9)	
≥3	2140 (23.4)	1282 (22.5)	858 (25.0)	
Age at menarche (years)				0.016
<13	1256 (13.7)	822 (14.4)	434 (12.6)	
≥13	7888 (86.3)	4884 (85.6)	3004 (87.4)	

### Disparities on specific knowledge of cervical cancer prevention between women who have ever and never screened for cervical cancer

As shown in Table 2, among the respondents, 89.7% had heard of cervical cancer (Q1), 73.2% were aware that cervical cancer could be prevented (Q3), and 68.4% recognized the benefits of regular cervical cancer screening (Q7). However, only 9.9% of women knew more than four of the seven risk factors for cervical cancer (Q2), and 10.0% knew more than four of the seven prevention measures for cervical cancer (Q4). Overall, 42.5% of women demonstrated a high level of knowledge (knowledge score ≥ 5). Women who had undergone cervical cancer screening had significantly higher proportions of correct answers for all seven items compared to those who had never screened before (*P* < 0.05). Furthermore, among women in the ever screened group, 58.3% exhibited a high level of knowledge, while only 33.0% of women in the never screened group were at a high knowledge level. Additionally, both the ever screened and never screened groups displayed a limited understanding of smoking as a risk factor for cervical cancer and the knowledge that avoiding smoking can prevent cervical cancer (*P* > 0.05).

**Table 2:** Knowledge of cervical cancer prevention among participants who have never and ever been screened for cervical cancer [correct responses, n(%)]

Knowledge-related questions	Total (*N* = 9144)	Never screened (*N* = 5706)	Ever screened (*N* = 3438)	*P*
Q1: Have heard of cervical cancer	8203 (89.7)	4905 (86.0)	3298 (95.9)	< 0.001
Q2: Knowledge of risk factors for Cervical Cancer (≥ 4)	902 (9.9)	456 (8.0)	446 (13.0)	< 0.001
Q2_1: Having multiple sexual partners	3457 (37.8)	1906 (33.4)	1551 (45.1)	< 0.001
Q2_2: Had sexual intercourse and children at a young age	1778 (19.4)	940 (16.5)	838 (24.4)	< 0.001
Q2_3: History of sexually transmitted diseases	2592 (28.3)	1326 (23.2)	1266 (36.8)	< 0.001
Q2_4: Smoking	826 (9.0)	500 (8.8)	326 (9.5)	0.245
Q2_5: History of HPV infection	1523 (16.7)	720 (12.6)	803 (23.4)	< 0.001
Q2_6: Aged 30–65 years old	1149 (12.6)	517 (9.1)	632 (18.4)	< 0.001
Q2_7: Long term use of oral contraceptives pills	905 (9.9)	520 (9.1)	385 (11.2)	0.001
Q3: Can cervical cancer be prevented	6693 (73.2)	3878 (68.0)	2815 (81.9)	< 0.001
Q4: Knowledge of how to Prevent Cervical cancer (≥ 4)	911 (10.0)	434 (7.6)	477 (13.9)	< 0.001
Q4_1: Getting vaccinated	3630 (39.7)	1932 (33.9)	1698 (49.4)	< 0.001
Q4_2: Having fewer sexual partners	1877 (20.5)	979 (17.2)	898 (26.1)	< 0.001
Q4_3: Regular cervical cancer screening	3116 (34.1)	1481 (26.0)	1635 (47.6)	< 0.001
Q4_4: Using condoms	988 (10.8)	461 (8.1)	527 (15.3)	< 0.001
Q4_5: Late marriage and late childbearing	494 (5.4)	274 (4.8)	220 (6.4)	0.001
Q4_6: Avoid smoking	696 (7.6)	428 (7.5)	268 (7.8)	0.607
Q4_7: Timely treatment of genital tract infections	1576 (17.2)	793 (13.9)	783 (22.8)	< 0.001
Q5: Have heard of HPV vaccines	5306 (58.0)	2947 (51.6)	2359 (68.6)	< 0.001
Q6: Know HPV vaccines can prevent cervical cancer	4805 (52.5)	2565 (45.0)	2240 (65.2)	< 0.001
Q7: Know the benefits of regular cervical cancer screening	6251 (68.4)	3161 (55.4)	3090 (89.9)	< 0.001
High Knowledge level (≥ 5)	3886 (42.5)	1883 (33.0)	2003 (58.3)	< 0.001

### Associations between knowledge level of cervical cancer and screening behaviors

We examined the associations between socio-demographic factors, knowledge level of cervical cancer and screening behaviors using univariate and multivariate logistic regression models (Table 3). The fully adjusted model results showed that region, area, age group, occupation, education level, marital status, gravidity and knowledge level were potential predictors to screening behaviors of women aged 20–49 years (*P* < 0.05). After adjusting for all socio-demographic factors, women with a high level of knowledge were more likely to have screening behaviors than those with a low knowledge level (OR = 2.91, 95% CI: 2.63–3.21).

**Table 3:** Logistic regression analysis on cervical cancer screening

Variable	Total			35－49 years old		
	Screened [*n*(%)]	Crude OR (95%CI)	AOR(95%CI)	Screened [*n*(%)]	Crude OR (95%CI)	AOR(95%CI)
**Region**						
Western	910 (29.9)	ref.	ref.	700 (36.6)	ref.	ref.
Central	1205 (40.3)	**1.58 (1.42**,** 1.76)**	**1.49 (1.32**,** 1.68)**	896 (47.6)	**1.57 (1.38**,** 1.79)**	**1.49 (1.29**,** 1.72)**
Eastern	1323 (42.5)	**1.74 (1.56**,** 1.93)**	**1.88 (1.66**,** 2.13)**	1001 (51.2)	**1.82 (1.60**,** 2.06)**	**2.11 (1.81**,** 2.44)**
**Area**						
Urban	1739 (38.5)	ref.	ref.	1282 (45.0)	ref.	ref.
Rural	1699 (36.7)	0.93 (0.85, 1.01)	**1.39 (1.23**,** 1.58)**	1315 (45.4)	1.02 (0.92, 1.13)	**1.96 (1.68**,** 2.28)**
**Age group (year)**						
20–24	70 (8.0)	ref.	ref.	-	-	-
25–29	273 (22.1)	**3.25 (2.46**,** 4.30)**	**1.58 (1.13**,** 2.19)**	-	-	-
30–34	498 (38.8)	**7.30 (5.58**,** 9.55)**	**3.32 (2.38**,** 4.64)**	-	-	-
35–39	542 (46.0)	**9.82 (7.49**,** 12.86)**	**5.13 (3.65**,** 7.21)**	542 (46.0)	ref.	ref.
40–44	961 (45.5)	**9.61 (7.42**,** 12.45)**	**6.05 (4.33**,** 8.46)**	961 (45.5)	0.98 (0.85, 1.13)	**1.24 (1.06**,** 1.46)**
45–49	1094 (44.5)	**9.21 (7.12**,** 11.91)**	**6.99 (5.00**,** 9.78)**	1094 (44.5)	0.94 (0.82, 1.08)	**1.44 (1.23**,** 1.68)**
**Occupation**						
Managerial and technical staff	708 (44.7)	ref.	ref.	463 (63.4)	ref.	ref.
Commercial/service personnel	595 (38.2)	**0.76 (0.66**,** 0.88)**	**0.78 (0.65**,** 0.93)**	446 (44.3)	**0.46 (0.38**,** 0.56)**	**0.69 (0.55**,** 0.87)**
Workers or farmers	1673 (39.9)	**0.82 (0.73**,** 0.92)**	0.93 (0.77, 1.12)	1364 (43.5)	**0.44 (0.38**,** 0.52)**	**0.77 (0.61**,** 0.98)**
Students	19 (6.7)	**0.09 (0.05**,** 0.14)**	0.70 (0.41, 1.22)	2 (33.3)	0.29 (0.05, 1.58)	0.79 (0.12, 5.10)
Unemployed	321 (26.8)	**0.45 (0.39**,** 0.53)**	**0.59 (0.48**,** 0.73)**	238 (34.8)	**0.31 (0.25**,** 0.38)**	**0.62 (0.47**,** 0.80)**
Others	122 (37.4)	**0.74 (0.58**,** 0.94)**	0.86 (0.65, 1.14)	84 (44.9)	**0.47 (0.34**,** 0.65)**	0.73 (0.51, 1.04)
**Education level**						
Primary school and below	508 (29.1)	ref.	ref.	483 (30.4)	ref.	ref.
Middle school	1241 (40.7)	**1.67 (1.48**,** 1.90)**	**2.23 (1.93**,** 2.56)**	1001 (46.7)	**2.01 (1.75**,** 2.30)**	**2.48 (2.13**,** 2.89)**
Senior high school or equivalent	768 (40.9)	**1.69 (1.47**,** 1.94)**	**2.57 (2.16**,** 3.06)**	584 (50.7)	**2.35 (2.01**,** 2.75)**	**3.11 (2.55**,** 3.80)**
College and above	921 (37.3)	**1.45 (1.27**,** 1.66)**	**3.53 (2.87**,** 4.35)**	529 (61.4)	**3.64 (3.06**,** 4.33)**	**4.66 (3.61**,** 6.01)**
**Monthly family income (RMB)**						
<3000	806 (33.1)	ref.	ref.	645 (38.1)	ref.	ref.
3000–4999	1045 (36.6)	**1.17 (1.04**,** 1.31)**	1.06 (0.93, 1.21)	820 (45.9)	**1.38 (1.20**,** 1.58)**	1.12 (0.97, 1.30)
5000–7999	908 (40.5)	**1.38 (1.22**,** 1.55)**	1.06 (0.92, 1.21)	680 (49.7)	**1.61 (1.39**,** 1.86)**	1.10 (0.93, 1.30)
≥8000	679 (42.2)	**1.48 (1.30**,** 1.68)**	0.93 (0.79, 1.10)	452 (50.3)	**1.64 (1.40**,** 1.93)**	0.88 (0.72, 1.07)
**Marital status**						
Unmarried	85 (8.4)	ref.	ref.	16 (30.2)	ref.	ref.
Married	3215 (41.3)	**7.68 (6.12**,** 9.64)**	**2.61 (1.83**,** 3.72)**	2456 (45.5)	**1.93 (1.07**,** 3.48)**	1.62 (0.84, 3.12)
Divorced/Widowed/Others	138 (39.8)	**7.21 (5.29**,** 9.82)**	**2.47 (1.63**,** 3.73)**	125 (42.2)	1.69 (0.90, 3.17)	1.68 (0.84, 3.36)
**Gravidity**						
0	166 (12.8)	ref.	ref.	53 (35.8)	ref.	ref.
1	1214 (42.6)	**5.04 (4.22**,** 6.03)**	**1.42 (1.08**,** 1.85)**	895 (48.5)	**1.69 (1.19**,** 2.39)**	1.27 (0.86, 1.89)
2	1200 (41.9)	**4.89 (4.09**,** 5.85)**	**1.56 (1.18**,** 2.04)**	916 (44.7)	**1.45 (1.03**,** 2.05)**	1.34 (0.90, 1.99)
≥3	858 (40.1)	**4.54 (3.78**,** 5.46)**	**1.61 (1.22**,** 2.12)**	733 (43.0)	1.35 (0.95, 1.92)	1.46 (0.98, 2.17)
**Age at menarche (years)**						
<13	434 (34.6)	ref.	ref.	281 (44.3)	ref.	ref.
≥13	3004 (38.1)	**1.16 (1.03**,** 1.32)**	1.00 (0.86, 1.16)	2316 (45.3)	1.04 (0.88, 1.23)	1.15 (0.95, 1.38)
**Knowledge level**						
Low level (< 5)	1435 (27.3)	ref.	ref.	1187 (34.1)	ref.	ref.
High level (≥5)	2003 (51.5)	**2.83 (2.60**,** 3.09)**	**2.91 (2.63**,** 3.21)**	1410 (62.3)	**3.21 (2.87**,** 3.58)**	**2.76 (2.45**,** 3.12)**

As a sensitivity analysis, logistic regression models were also performed among women aged 35–49 years. Region, area, age group, occupation, education level and knowledge level were also associated with cervical cancer screening behaviors in fully adjusted models (*P* < 0.05). After adjusting for socio-demographic factors, it was also found that high knowledge level of cervical cancer was significantly associated with screening behaviors (OR = 2.76, 95% CI: 2.45–3.12).

## Associations between knowledge score of cervical cancer and screening behaviors in different regions and areas

We further investigated the associations between knowledge scores of cervical cancer and screening behaviors in different regions (Table 4) and different areas (Table 5). The results showed that among 20-49-year-old women, there was a significant upward trend in the screening rate with the knowledge score increased (P_trend_<0.001) in all three regions (western, central, and eastern). In cases where the knowledge score was higher than 6 scores, the screening rate exceeded 50% in all regions. Compared to women in ≤ 1 score group in the western region, the screening rate of women with higher than 6 scores significantly increased (OR = 19.62, 95% CI: 12.39–31.04). Similarly, the positive associations were also found in the eastern and central regions (OR = 10.09, 95% CI: 6.76–15.06 for central region; OR = 5.23, 95%CI: 3.62–7.56 for eastern region).

In the 35-49-year-old women, similar results were observed. There was a significant increase in the screening rate of cervical cancer (P_trend_<0.001) as the knowledge score increased. Among participants with a knowledge score ≥ 6 scores, the screening rate exceeded 70% in the eastern region, and was above 65% in both the western and central regions.

**Table 4:**
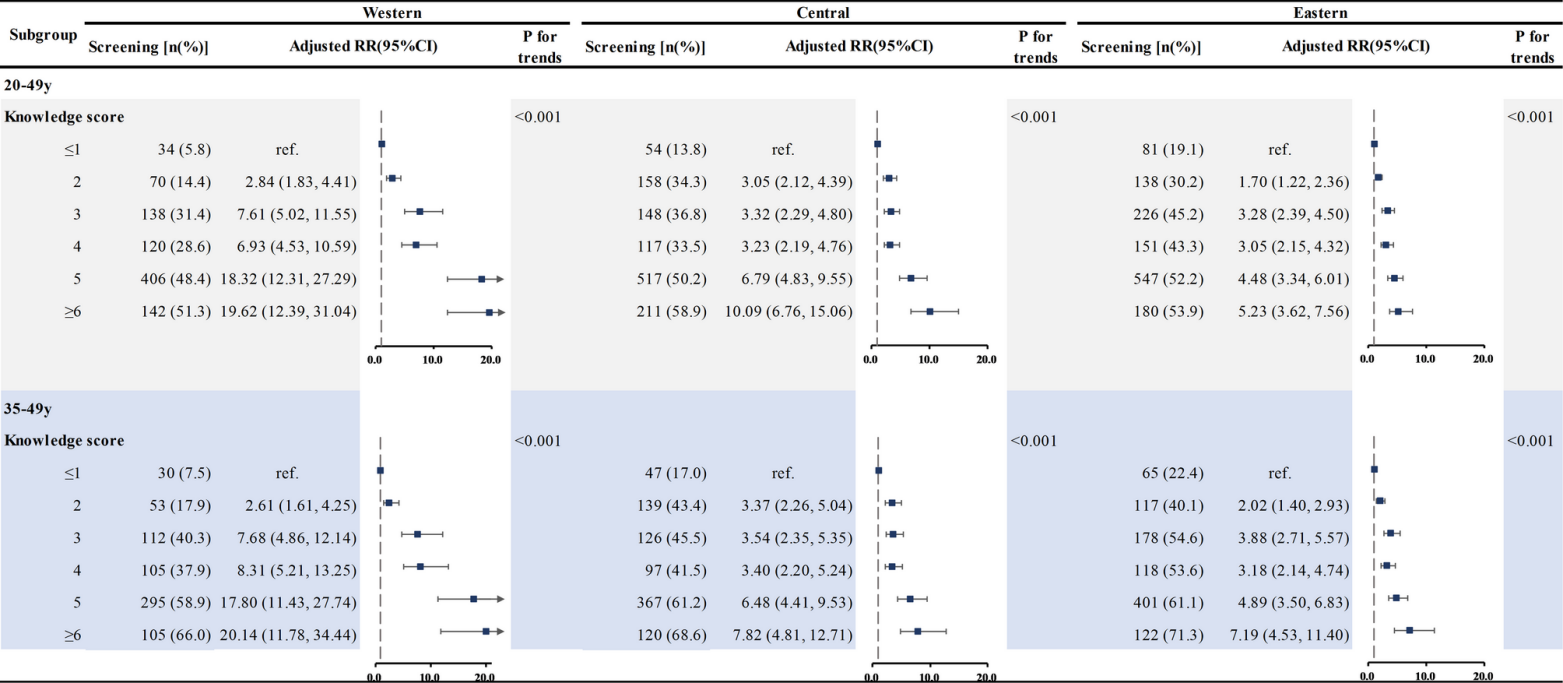
Association of knowledge score with cervical cancer screening in different regions

*Note* All models adjusted for area, age group, occupation, education level, monthly family income, marital status, gravidity and age at menarche

**Table 5:**
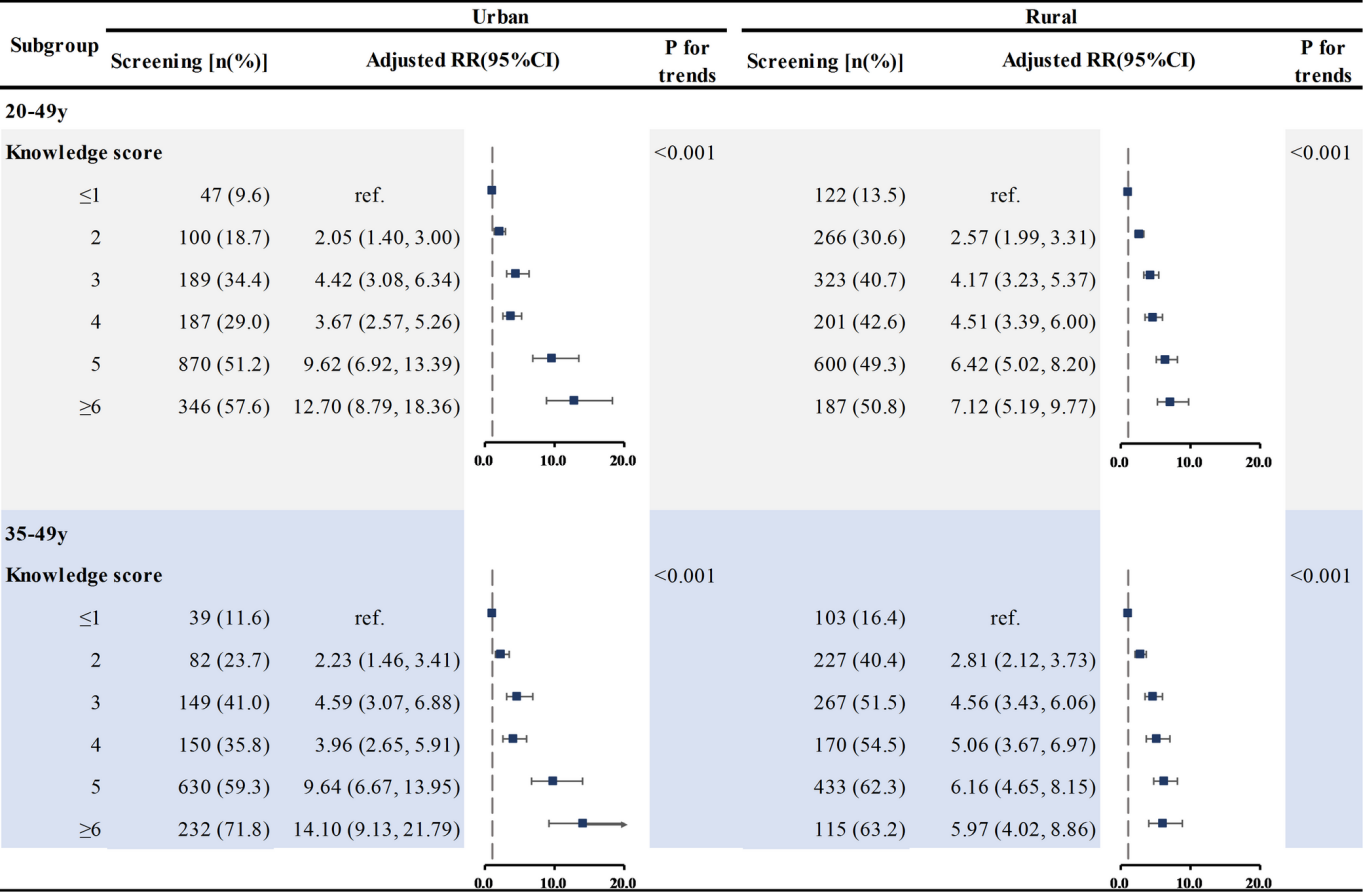
Association of knowledge score with cervical cancer screening in urban and rural areas

*Note* All models adjusted for region, age group, occupation, education level, monthly family income, marital status, gravidity and age at menarche

## Discussion

Based on this cross-section study, we found that 37.6% of women aged 20–49 years in China reported experience of cervical cancer screening. The findings from our study suggested that the level of cervical cancer knowledge was relatively low, with less than half of women demonstrating a high knowledge level (score ≥ 5). The proportion of high knowledge level of women in ever screened group was significantly higher than that in never screened group. After adjusting for socio-demographic factors, women with a high level of knowledge were more likely to have screening behaviors than those with a low knowledge level, both in the east, central and western region, and also in urban and rural area.

Our findings suggested that cervical cancer screening rates among women in 2018 in China had not yet reached the target of 70% in 2030. Furthermore, the screening rates varied significantly among different age groups. Screening rates were lower for women aged 20–34 years old at less than 40%, while higher rates of more than 45% for women aged 35–49 years old. This may be related to the requirements of the age range (35 to 64 years old) in China’s cervical cancer screening work plan, which was based on cost-effectiveness considerations. According to the national survey data from China Chronic Disease and Nutrition Surveillance in 2018–2019, cervical cancer screening coverage reached 43.4% in women aged 35–44 years, and 36.8% in women aged 35–64 years [5]. The screening rates were similar with our results, and also showed that screening rates were still at a lower level in rural areas as well as central and western regions. Cervical cancer screening rates have improved over the past few years compared to the results in 2015 [16], which may be related to the relevant policies implemented on screening at the national level. Nonetheless, screening coverage in China was still obviously lower than those in high-income countries, such as U.S. (over 80%) [17]. Therefore, improving cervical cancer screening rates and adherence remains an important issue in China at present. Multiple factors, such as region, age group, occupation, education level, family income, marital status, and gravidity, were identified as potential predictors of cervical cancer screening behaviors, which were similar with previous studies [7, 18, 19]. This demonstrates the socio-economic disparities in China, indicating that people with lower socioeconomic status may not have equal access to social services, even the organizational screening services were free. So, they are likely to be the main target population when increasing screening rates.

It is generally accepted that the knowledge level motivates the search for relevant health practices for cervical cancer prevention [20]. The knowledge level of cervical cancer among women in China is relatively low, yet in our survey. The measurement tool of cervical cancer knowledge was varied in different studies [21–23], and it is difficult to compare with other studies directly. While most women have heard of cervical cancer, they have limited knowledge of its risk factors and preventive measures. Specifically, only 9.9% of women knew more than four out of the seven risk factors for cervical cancer, and merely 10.0% were familiar with more than four out of the seven prevention measures. The results showed a relatively high level of the awareness of sexual risk factors for cervical cancer, but lower level about non-sexual risk factors such as smoking, long-term use of oral contraceptive pills and age, which were similar with the previous study [24]. This highlights the importance of targeted educational interventions to improve knowledge in these areas and promote informed decision-making regarding cervical cancer prevention and early detection.

The most important findings from our study suggest that women with high knowledge level of cervical cancer were more likely to have screening behavior (OR = 2.91, 95%CI: 2.63–3.21), and higher knowledge scores are associated with increased screening behaviors in different regions and areas (P_trend_ < 0.001). The results suggest that lack of knowledge may be a barrier to screening. For women aged 30-59-years, the screening rate could reach the target of 70% while the knowledge score was 6 or greater. This disparity emphasizes the need to bridge the gap between knowledge and screening behavior among women. Previous studies have also shown that health education interventions are effective in cervical cancer prevention [25]. Therefore, this study suggested that improving knowledge and awareness of cervical cancer may contribute to the promotion of screening behaviors and the prevention of cervical cancer.

Several limitations in this study should be noticed. Firstly, the data was collected from districts/counties in provincial capitals, which may overestimate the screening rates and knowledge levels within these provinces. However, it does not affect the conclusion that there was a positive association between knowledge and behavior. Secondly, the investigation of cervical cancer screening behaviors in this study was self-reported, which may lead to a certain degree of recall bias. Moreover, the knowledge of cervical cancer prevention was evaluated by a self-designed questionnaire, which might have potential implications on the comparability with other studies. To minimize the impact on comparability, we have made efforts to align our questionnaire with existing published guidelines on cervical cancer prevention. Thirdly, due to the cross-sectional design of this study, the causal relationships between knowledge and screening behaviors cannot be inferred. Fourthly, due to the study design, this study did not assess the knowledge level among women aged 50–64 years, therefore no analysis was conducted for this age group. The study mainly focused on reproductive-age women who were under 50 years old. Additionally, since there is a national routine cervical cancer screening program for women over 35 years old in China, the study conducted a separate analysis to explore the associations within the age group of 35–49 years. Future studies are encouraged to explore how to improve cervical cancer screening behaviors and overcome the existing barriers.

## Conclusions

Overall, our study showed that the screening rate and knowledge level of cervical cancer were relatively low among women. Women with a higher score of knowledge were more likely to have screening behaviors, regardless of the region or area. Our study indicates that it is necessary to enhance the knowledge level and health literacy regarding cervical cancer through intervention measures, and to bridge the gap between knowledge and behavior in order to promote regular cervical cancer screening and improve women’s health in China.

## Data Availability

The datasets used and/or analysed during the current study are available from the corresponding author on reasonable request.
